# Investigation of Drug–Polymer Compatibility Using Chemometric-Assisted UV-Spectrophotometry

**DOI:** 10.3390/pharmaceutics9010007

**Published:** 2017-01-16

**Authors:** Amir Ibrahim Mohamed, Amr Mohamed Elsayed Abd-Motagaly, Osama A. A. Ahmed, Suzan Amin, Alaa Ibrahim Mohamed Ali

**Affiliations:** 1 Pharmaceutics and industrial pharmacy, Military Medical Academy, Cairo 00202, Egypt; amr.abdmotagaly@gmail.com; 2 Department of Pharmaceutics and Industrial Pharmacy, Faculty of Pharmacy, King Abdulaziz University, Jeddah 21589, Saudi Arabia; osama712000@gmail.com; 3 Medical services Department, Cairo 00202, Egypt; dr.suzanamin@gmail.com; 4 Faculty of Pharmacy, Cairo University, Cairo 11562, Egypt

**Keywords:** clindamycin HCl, sodium alginate, chitosan, partial least squares regression (PLS), drug–polymer compatibility

## Abstract

A simple chemometric-assisted UV-spectrophotometric method was used to study the compatibility of clindamycin hydrochloride (HC1) with two commonly used natural controlled-release polymers, alginate (Ag) and chitosan (Ch). Standard mixtures containing 1:1, 1:2, and 1:0.5 *w*/*w* drug–polymer ratios were prepared and UV scanned. A calibration model was developed with partial least square (PLS) regression analysis for each polymer separately. Then, test mixtures containing 1:1 *w*/*w* drug–polymer ratios with different sets of drug concentrations were prepared. These were UV scanned initially and after three and seven days of storage at 25 °C. Using the calibration model, the drug recovery percent was estimated and a decrease in concentration of 10% or more from initial concentration was considered to indicate instability. PLS models with PC3 (for Ag) and PC2 (for Ch) showed a good correlation between actual and found values with root mean square error of cross validation (RMSECV) of 0.00284 and 0.01228, and calibration coefficient (*R*^2^) values of 0.996 and 0.942, respectively. The average drug recovery percent after three and seven days was 98.1 ± 2.9 and 95.4 ± 4.0 (for Ag), and 97.3 ± 2.1 and 91.4 ± 3.8 (for Ch), which suggests more drug compatibility with an Ag than a Ch polymer. Conventional techniques including DSC, XRD, FTIR, and in vitro minimum inhibitory concentration (MIC) for (1:1) drug–polymer mixtures were also performed to confirm clindamycin compatibility with Ag and Ch polymers.

## 1. Introduction

Drug–excipient compatibility is one of the most important parameters to be considered during pre-formulation studies, as it can alter the physicochemical properties and bioavailability of the drugs involved. Especially for antibiotics, it is necessary to determine the drug compatibility or incompatibility either in oral preparations or in parenteral admixtures before they appear in the clinical setting. To develop effective, safe and stable formulation drug–excipient compatibility is an important process in pharmaceutical product development but the cost and time associated with this process represent a major challenge and make this type of predictability technique more desirable [1]. Despite of the importance of drug–excipient compatibility tests, there is no universally accepted protocol for this purpose. The most frequently used methods for the physico-chemical investigation include thermal analysis (DSC, DTA, DTG, ITC), spectroscopic methods (FT-IR, X-ray diffraction, NMR), and chromatographic methods (LC, LC-MS/MS, HPLC) [2]. Some of the reported methods have poor predictive values, while others are tedious and time consuming. Therefore, simple, effective, rapid and reliable methods using low-cost equipment seem to be necessary.

Spectroscopic techniques are generally fast, with the analysis taking from a few seconds to a few minutes, and can generate large amounts of data for each sample analyzed. This data can be said to consist of two parts: information and noise. The information part of the data is what eventually leads to knowledge about the sample, while the noise is a non-information part. This is where chemometrics comes in. Chemometric tools can extract the information from large sets of data and markedly increase the quality of the spectral information [3]. The use of spectroscopic techniques coupled with chemometric tools offers many advantages in qualitative and quantitative spectroscopic analysis. The methods generally become more robust, precise, and less sensitive to spectral artefacts. Nowadays, various applications performed by means of UV-Vis spectrophotometry and chemometric techniques have been reported, such as simultaneous determination of drugs in commercial combined formulations [4,5], as well as purity and quantitative analysis of raw materials and active ingredients in the manufacturing of pharmaceuticals, cosmetics, foods and beverages [6,7,8,9]. In addition, these techniques have been applied for stability studies and evaluation of photo-degradation kinetics under different reaction conditions [10]. To our knowledge, there have not yet been reports involving the use of chemometric-assisted UV-spectrophotometry for drug–excipient compatibility studies.

In this study, a simple chemometric-assisted UV-spectrophotometric method was used to research the compatibility of a model antibiotic drug (clindamycin hydrochloride) with two commonly used natural bio-degradable controlled-release polymers, Alginate and Chitosan. Standard drug–polymer mixtures containing 1:1, 1:2, and 1:0.5 *w*/*w* drug–polymer ratios at a concentration range of 0.008–0.14 mg/mL were prepared and UV scanned in the range of 190–250 nm. The UV calibration model was developed with partial least square (PLS) regression analysis for each polymer separately. Then, test drug–polymer mixtures containing 1:1 *w*/*w* drug–polymer ratios with different sets of drug concentrations were prepared and UV spectra were collected initially and after three and seven days of storage at temperatures of 25 °C [11]. Using the calibration model of each polymer, the drug recovery percent was estimated and a decrease in concentration of 10% or more from initial concentration was considered to indicate instability [12]. In addition, conventional techniques including DSC, XRD, FTIR, and in vitro minimum inhibitory concentration (MIC) for (1:1) drug–polymer mixtures were also performed to confirm the results obtained.

## 2. Materials and Methods

### 2.1. Materials

Clindamycin hydrochloride was purchased from Sigma-Aldrich Chemie GmbH (Riedstrasse, Germany). Sodium alginate, molecular weight of 216.12 g/mol was purchased from Sas chemicals (Mumbai, India). Chitosan, molecular weight of 10,000–30,000 Da was obtained from Srividya Enterprises (Ratnagiri, India). Acetic acid was purchased from El-Nasr Chemicals Co. (Cairo, Egypt). All other chemicals were of analytical grade.

### 2.2. Methodology

#### 2.2.1. UV-Spectroscopic Studies

Absorbance spectra were collected with Agilent Cary 60 UV-Vis spectrometer (Agilent, Richardson, TX, USA) equipped with computer software package UV Agilent Cary 60 version 5.0.0.999, using a 10 mm path length quartz cell over the wavelength range from 190 to 1100 nm.

##### Preparation of Standard Stock Solutions

Stock solutions of the single compounds (clindamycin, alginate and chitosan) were prepared to get standard stock solutions with a concentration of 10 mg/mL. For the drug, an accurately weighed amount of clindamycin HCl equivalent to 100 mg of clindamycin was transferred into a 10 mL volumetric flask and diluted to volume with 1 M phosphate buffer of pH 7.5. Sodium alginate was dissolved in distilled water while chitosan was dissolved in a 0.5% acetic acid solution with a magnetic stirrer at 100 rpm [13].

##### Selection of Appropriate Wavelength Range

The absorbance spectrum of free clindamycin standard solution with a concentration of 4 mg/mL was scanned from 190 to 1100 nm against phosphate buffer as blank. The wavelength range with maximum sensitivity of measured absorbance (absorption maxima) and minimum interference from instrumental factors such as signal-to-noise ratio was selected.

##### Selection of Linear Concentration Range

Free clindamycin standard solutions with a concentration range of 0.0075–4 mg/mL were scanned within the selected wavelength rage and the multi-point calibration curve was plotted. The concentration range that shows linear response to drug concentration was selected for calibration model setting.

##### Preparation of Standard Calibration Drug–Polymer Mixtures

A calibration set of 15 standard mixture solutions containing five drug concentrations (according to its linear calibration range) and three polymer level ratios (1:1, 1:2 and 1:0.5 *w*/*w* drug:polymer ratio), was made in 10 mL volumetric flasks from the standard stock solutions using a phosphate buffer with pH 7.5 as diluent for each polymer.

##### Preparation of Test Drug–Polymer Mixtures

An independent test set of five standard mixture solutions containing five drug concentrations (different from those used in the calibration set but still within the linear calibration range) and one polymer level ratio (1:1 *w*/*w* drug:polymer) was made in 10 mL volumetric flasks from the standard stock solutions using a phosphate buffer with pH 7.5 as a diluent.

##### Data Collection

Each solution sample was prepared in duplicate and UV spectra of the calibration drug–polymer mixtures and the test drug–polymer mixtures were collected over the selected wavelength range at room temperature (25 °C). Each sample was run for three scans with a bandwidth of 1 nm, scan rate of 1 nm/s, time response of 1 s, and data interval of 1.0 point/nm. The test drug–polymer mixtures were stored at room temperature and re-scanned after three and seven days. For the chitosan test mixture, samples were re-scanned after filtration via 3K Amicon Ultra-2 centrifugal filter device using 40° fixed angle centrifuge for 60 min at 7500× *g*.

##### Chemometric Analysis

Spectral data collected were imported into Essential FTIR™ software (15th Edition, Operant LLC, Madison, WI, USA) for pre-treatment and chemometric modeling. Partial least square (PLS) regression analysis was applied to explore data and to develop calibration models for both polymers. The performance of the UV calibration model was evaluated with leave-one out cross validation (CV), in which one sample is removed from the calibration set at a time and the same sample is predicted by using the calibration model built with the remaining samples. The appropriate number of PLS factors (principal components) for each model was chosen by adopting the root mean square error of cross validation (RMSECV), and the calibration coefficient (*R*^2^). The best prediction ability of the models corresponds to the lowest RMSECV and highest calibration coefficient (*R*^2^) [14].

##### Recovery Analysis

Clindamycin concentrations in the polymer test mixtures were assessed using the calibration model of each polymer at zero time and after three and seven days of storage at temperatures of 25 °C. The drug concentrations at time zero were defined as 100%, and subsequent sample concentrations were expressed as a percentage of these initial concentrations. Decreases in concentration of 10% or more from initial concentration were considered to indicate instability.

#### 2.2.2. Physico-Chemical Analysis

##### Preparation of Drug–Polymer Physical Mixtures and Blend Films

Physical mixtures of 1:1 *w*/*w* were prepared by mixing 50 mg clindamycin HCl with 50 mg of each polymer separately. Drug–polymer blend films were formed by using a simple solvent evaporation casting method [15]. Distilled water was used as solvent for water-soluble substances (clindamycin and sodium alginate), while 0.5% acetic acid solution was used for the chitosan polymer. For both clindamycin-alginate films and clindamycin-chitosan films, 5 mL of drug solution (5 mg/mL) was mixed with 5 mL of polymer solution (5 mg/mL) to get a mixture of 1:1 *w*/*w* drug:polymer ratio. The mixture obtained was cast to glass petri-dishes and evaporated at room temperature for 48 h.

##### Differential Scanning Calorimetry (DSC) Thermal Analysis

The DSC thermograms for each of the following (drug alone, physical mixture, and drug–polymer blend films) were collected on a Perkin Elmer analyzer (Perkin Elmer, Waltham, MA, USA) equipped with computer software program at a scanning rate of 10 °C·min^−1^. Each 10 mg sample was weighed and sealed in a flat bottomed aluminum pan, then heated from 30–450 °C in an atmosphere of nitrogen.

##### X-ray Diffraction Crystallography (XRD)

X-ray spectra for drug, physical mixture and drug–polymer blend films were measured using Philips X-ray diffraction equipment model PW/1710 (Philips, Amsterdam, The Neitherlands) with copper tube anode, 40 Kv voltage, and 35 mA current generator. The diffraction patterns were obtained over a range of 2θ angles from 2°–50° at an angular speed of 0.02° per second.

##### Fourier Transform Infrared (FTIR) Spectral Analysis

Samples (clindamycin, physical mixtures, and blend films) were scanned at the functional group region (4000–1500 cm^−1^) using a Shimadzu FT-IR spectrometer (Shimadzu, Kyoto, Japan). The samples were mixed with 200 mg of KBr and the obtained disks were analyzed at a resolution of 4 cm^−1^. Each spectrum was the mean of 16 consecutive scans of the same sample.

#### 2.2.3. Effect of Polymers on Clindamycin Anti-Bacterial Activity against *Staphylococcus aureus*

##### Bacterial Isolates and Culture Conditions

*Staphylococcus aureus* ATCC 6538 was obtained from the Microbiological Resources Centre (MIRCEN, Cairo, Egypt). Primary isolation was performed on selective nutrient agar supplemented with sheep blood 10% (*v*/*v*). Following primary selective isolation, *Staphylococcus aureus* bacterial cells were identified according to colony morphology and gram stain. Five drops of *Staphylococcus aureus* suspension was prepared in sterile distilled water to opacity of 0.5 McFarland standards (1.5 × 10^8^ colony forming unit/mL) [16].

##### Preparation of Sterile Stock Solutions (Drug or Polymer)

All stock solutions were sterilized by filtration with Millipore bacterial filters (0.22 μ membrane syringe filter), transferred into sterile tube with rubber cap (blood collection tube), and stored away of light and heat. For water-soluble clindamycin and sodium alginate, 102.4 mg was accurately weighed and placed in a 10 mL volumetric flask and completed to 10 mL with distilled water to get a concentration of 10.24 mg/mL (10,240 μg/mL). For chitosan, 102.4 mg was placed in a 10 mL volumetric flask and diluted with 0.5% acetic acid solution.

##### Preparation of Working Dilutions for Agar Susceptibility Tests

A conventional two-fold dilution scheme was used to prepare different concentration ranges of antimicrobial agent (drug or polymer). Agar dilution susceptibility test was used as a comparative testing to determine the minimum inhibitory concentration (MIC) of clindamycin, polymer (if any), and clindamycin-polymer 1:1 mixture blend. In a 50 mL sterile falcon tube, using sterile pipettes, 1 mL volume of the prepared dilution series was added to 19 mL of sterile molten agar (nutrient agar with 10% sheep blood), mixed thoroughly, and poured into 90 mm sterile Petri dishes to get a final concentration in medium of 0.03–512 μg/mL. Blends of clindamycin-polymer were prepared by mixing 1 mL of each drug dilution with 1 mL of polymer of the same concentration (μg/mL) to get a mixture of 1:1 drug/polymer solution, then added to 18 mL of molten agar (20 mL total volume) to keep a final drug concentration of 0.03–512 μg/mL. In addition, distilled water was used as control test and 0.5% acetic acid solution as solvent control.

##### Bacterial Growth Inhibition Assay (Agar Dilution Susceptibility Method)

Five drops of *Staphylococcus* *aureus* suspension (equivalent to a no. 0.5 McFarland standard) was inoculated on agar plates containing different concentrations of drug, polymer, or a 1:1 blend. After 24 h of incubation 37 °C, Plates were examined visually, and the lowest concentration of antibiotic showing complete inhibition of bacterial growth was recorded as MIC.

## 3. Results and Discussion

### 3.1. UV-Spectroscopic Studies

#### 3.1.1. Selection of Wavelength Range

**Figure 1:**
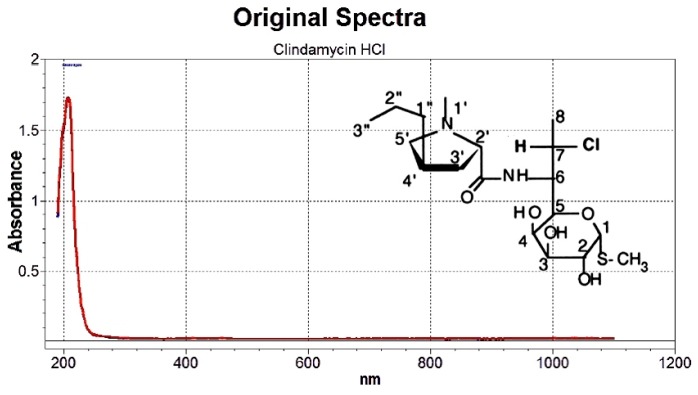
UV spectrum of clindamycin HCl in phosphate buffer pH 7.5.

The spectrum for free clindamycin is shown in Figure 1. It is clear that clindamycin exhibits a significant absorption signal in the 190–250 nm region which is mainly related to electronic transitions involving pi (π), non-bonding (*n*) electrons of the carbonyl group, and hetero-atoms such as oxygen, nitrogen, or sulfur present in clindamycin molecules [3]. The wavelengths over 250 nm were discarded due to the absence of absorption signals and because any absorbance values obtained at these wavelengths would have introduced a significant amount of noise in the calibration matrix, thereby decreasing the chemometric model precision [17].

#### 3.1.2. Selection of Concentration Range

**Figure 2:**
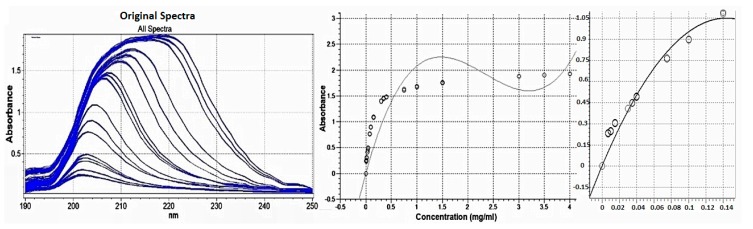
Multi-point calibration curve of clindamycin HCl at concentration range of 0.0075–4 mg/mL.

In accordance with Beer’s law, a linear relationship should exist between the absorbance and the concentration, assuming the path length is kept constant. A multi-point calibration curve was performed using Essential FTIR™ software (Figure 2). The response of the drug was found to be linear in the concentration range of 0.008–0.14 mg/mL with a correlation coefficient of 0.982.

#### 3.1.3. Preparation of Calibration and Test Drug–Polymer Mixtures

Fifteen standard calibration mixtures were prepared for each polymer (alginate and chitosan) containing five drug concentrations (0.008, 0.02, 0.06, 0.1, and 0.14 mg/mL) and three polymer level ratios (1:1, 1:2 and 1:0.5 *w*/*w* drug:polymer ratio) using a phosphate buffer at pH 7.5 as diluent. Five test mixtures were also prepared for each polymer containing five different drug concentrations (0.01, 0.03, 0.05, 0.08, and 0.12 mg/mL) and one polymer level ratio (1:1 *w*/*w* drug:polymer) using the same stock solutions. Each concentration was prepared in duplicate and the spectrophotometric readings were measured at a wavelength range of 190–250 nm by using phosphate buffer at a pH 7.5 as a blank.

#### 3.1.4. Chemometric Analysis

**Figure 3:**
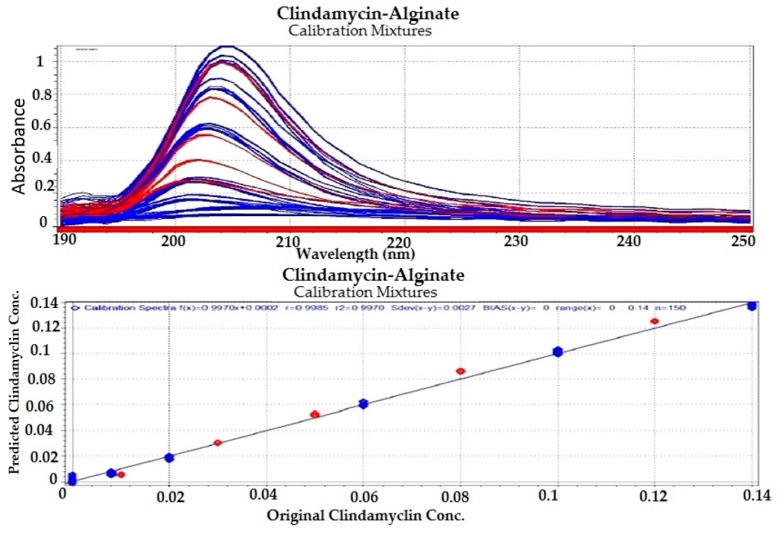
Regression plot for clindamycin concentration predicted by the model versus the known values in the calibration drug–alginate mixtures. Blue color for calibration set (C-set), while red color for validation set (V-set).

**Figure 4:**
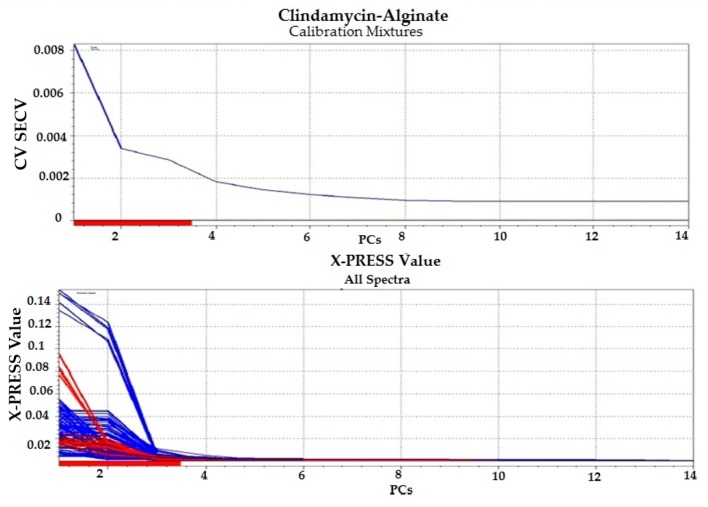
Selection of appropriate number of partial least square (PLS) components for clindamycin-alginate calibration mixtures. Blue color for calibration set (C-set), while red color for validation set (V-set).

**Figure 5:**
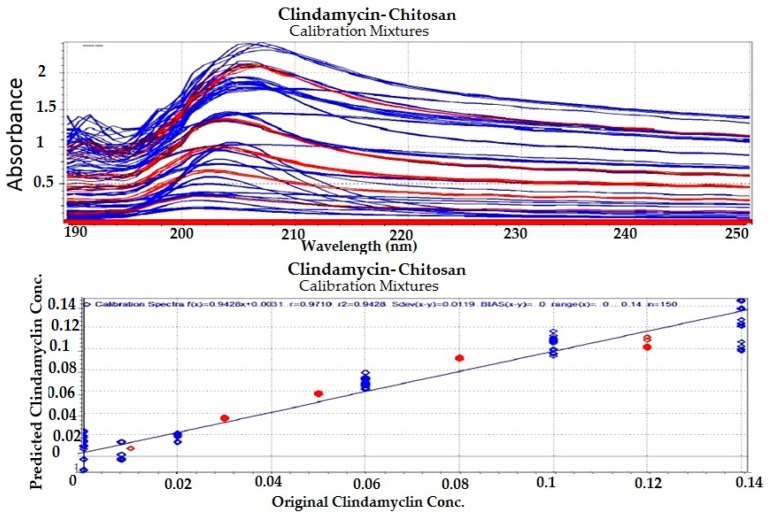
Regression plot for clindamycin concentration predicted by the model versus the known values in the calibration drug–chitosan mixtures. Blue color for calibration set (C-set), while red color for validation set (V-set).

**Figure 6:**
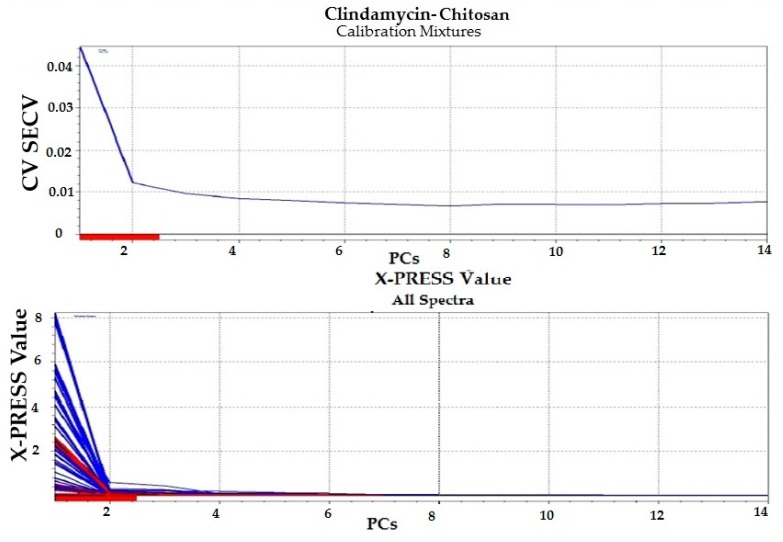
Selection of appropriate number of PLS components for clindamycin-chitosan calibration mixtures.

Partial least squares (PLS) regression modeling was used to develop the regression models for the drug–polymer calibration mixtures. Because UV spectral data are relatively noise-free, only mean-centering pre-treatment was employed, then the spectral data were input into the PLS software (Figure 3, Figure 4, Figure 5 and Figure 6). In developing a regression model, selecting the proper number of PLS components to use is an important consideration. Using too few PLS components will result in sub-optimal predictive capability, while using too many PLS components will result in over-fitting the model, incorporating noise into the model which has no predictive capability. As a result, use of too many PLS components will also reduce the predictive capability of the model [18]. Figure 4 and Figure 6 explain how we can get the optimum PC numbers using “X-PRESS plot” that shows which PCs have an influence on the spectra reconstruction. When the X-PRESS values are small and do not change significantly anymore this indicates that additional PCs do not improve the reconstruction of the spectra. These additional PCs contain little additional useful information. The smallest number of PCs that still shows changes should be selected. In this study, models with 3 PLS components (for alginate mixtures) and 2 PLS components (for chitosan mixtures) had better predictive capabilities (lower RMSECV), and more linear calibration plots. Table 1 shows the statistical parameters from leave-one out cross validation (CV) of chemometric models for drug–polymer calibration mixtures in the range of 190–250 nm. For the alginate calibration set, the linear plot has a correlation coefficient of 0.996, an offset of 0.0000774, and RMSECV of 0.00284, while for the chitosan calibration set, the linear plot has *R*^2^ of 0.942, an offset of 0.0052874, and RMSECV of 0.01228. A perfect model would have a *R*^2^ of 1, an offset of 0, and the lowest possible RMSECV value [17].

#### 3.1.5. Recovery Analysis

The results of clindamycin recovery studies from the polymer test mixtures are summarized in Table 2. Less than 5% loss of drug content with respect to initial concentration was observed for clindamycin-alginate test mixtures even after seven days of storage. On the other hand, chitosan test mixtures showed noticeable loss of drug content, especially after seven days of storage (19.7%). The colloidal chitosan turbidity developed due to low solubility of polymer in phosphate buffer pH 7.5 and may have interfered and/or adsorbed free clindamycin in the drug–polymer test mixture. After filtration, chitosan test mixtures showed an improvement in drug recovery and less than 8.7% loss of drug content was observed after seven days which indicates clindamycin stability in the filtered test mixture.

### 3.2. Physico-Chemical Analysis

#### 3.2.1. DSC Thermal Analysis and X-ray Diffraction

**Figure 7:**
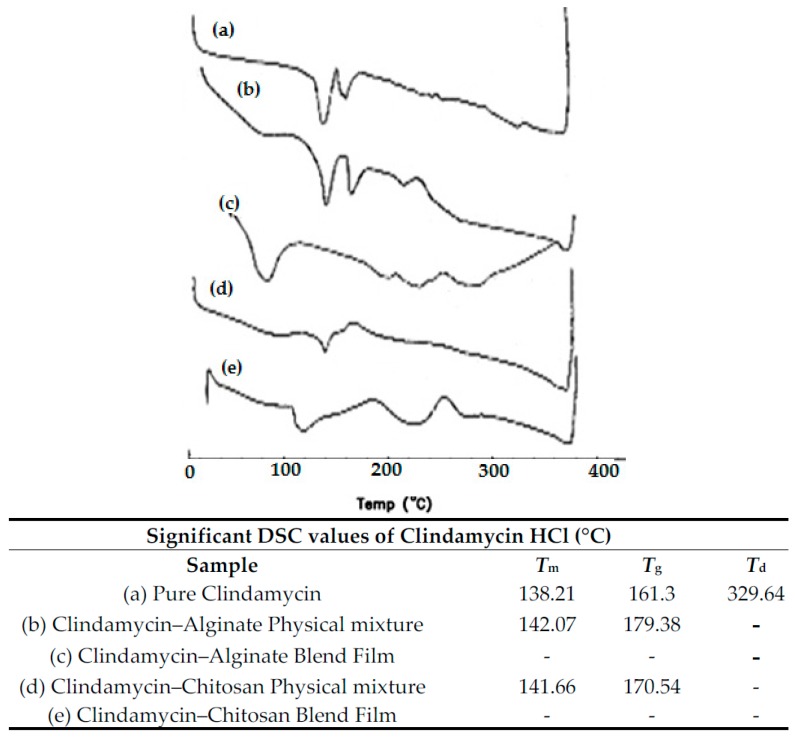
Significant data and DSC curves of (a) pure clindamycin; (b) clindamycin-alginate physical mixture; (c) clindamycin-alginate blend films; (d) clindamycin-chitosan physical mixture; (e) clindamycin-chitosan blend films.

**Figure 8:**
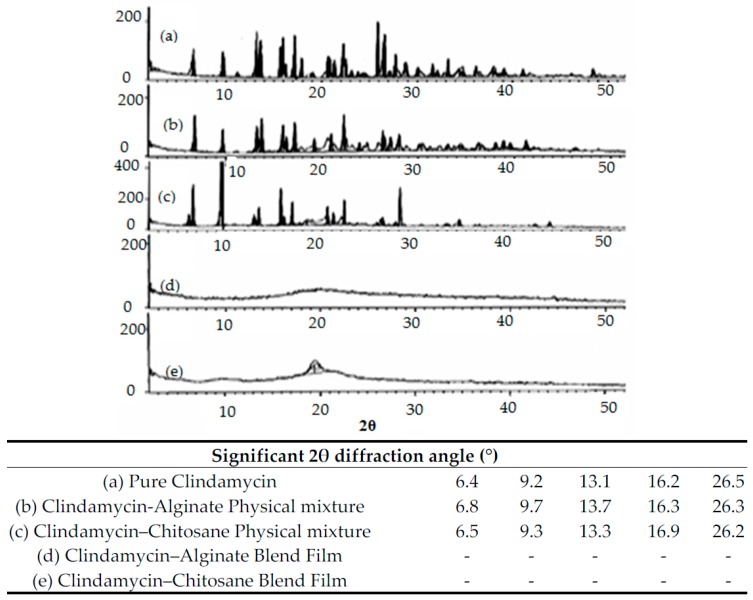
Significant data and X-ray curves of (a) pure clindamycin; (b) clindamycin-alginate physical mixture; (c) clindamycin-alginate blend films; (d) clindamycin-chitosan physical mixture; (e) clindamycin-chitosan blend films.

DSC and X-ray analysis allowed the evaluation of the drug’s physical state changes and the possible interactions between the drug and used excipients [2]. Figure 7 shows a DSC thermogram of pure clindamycin HCl that has three endothermic transitions. The characteristic sharp endothermic transition (*T*_m_) around 138.41 °C is mainly related to clindamycin’s melting point (142 °C, as previously reported by [19]). Another endothermic peak appeared at 161.3 °C which could be related to clindamycin’s glass transition temperature (*T*_g_) [20]. Finally, a mild endothermic peak also appeared at 329 °C, which may be attributed to drug decomposition. It is clear that clindamycin’s Tm peak appears in all drug–polymer physical mixtures with minor changes in position, indicating that neither polymer used affected the crystalline nature of clindamycin HCl and the absence of interaction at solid state. The DSC results of clindamycin-polymer films shows the disappearance of the characteristic *T*_m_ peak of the drug which can be explained by the transformation of clindamycin from an unstable crystalline form to its stable amorphous form during dissolving and re-precipitation processes [21]. Figure 8 shows the X-ray pattern of pure clindamycin with numerous sharp and distinct peaks at 2θ angle of 6.4°, 9.2°, 13.1°, 16.2°, 26.5° indicating crystalline nature of drug [13]. In both physical mixtures, clindamycin peaks were found, while blend films showed absence of the crystalline peaks of drug, further confirming the DSC results.

#### 3.2.2. Fourier Transform Infrared (FTIR) Spectral Analysis

**Figure 9:**
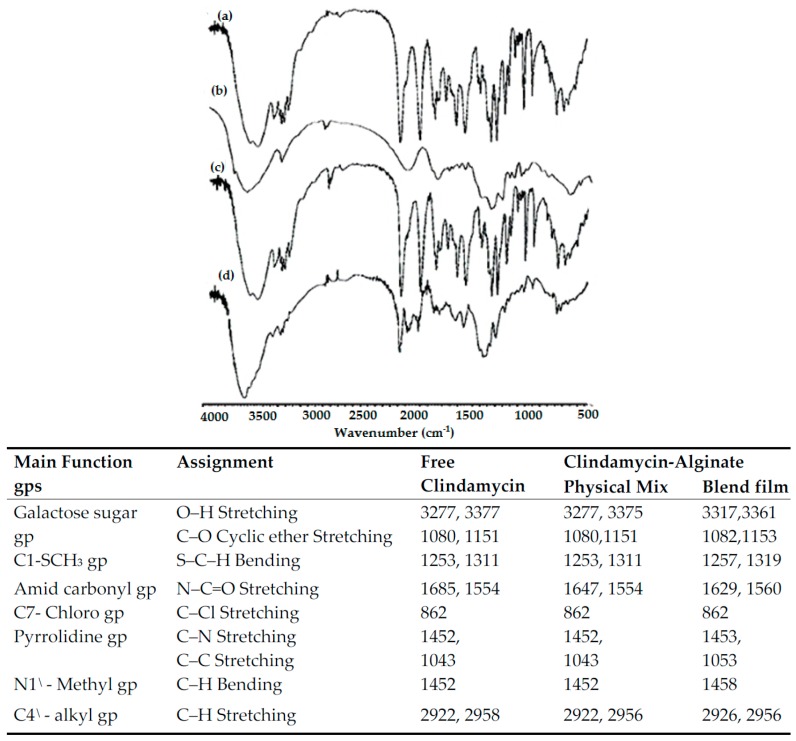
Most effective FTIR bands and spectra of: (a) pure clindamycin; (b) alginate alone; (c) clindamycin-alginate physical mixture; (d) clindamycin-alginate blend film.

**Figure 10:**
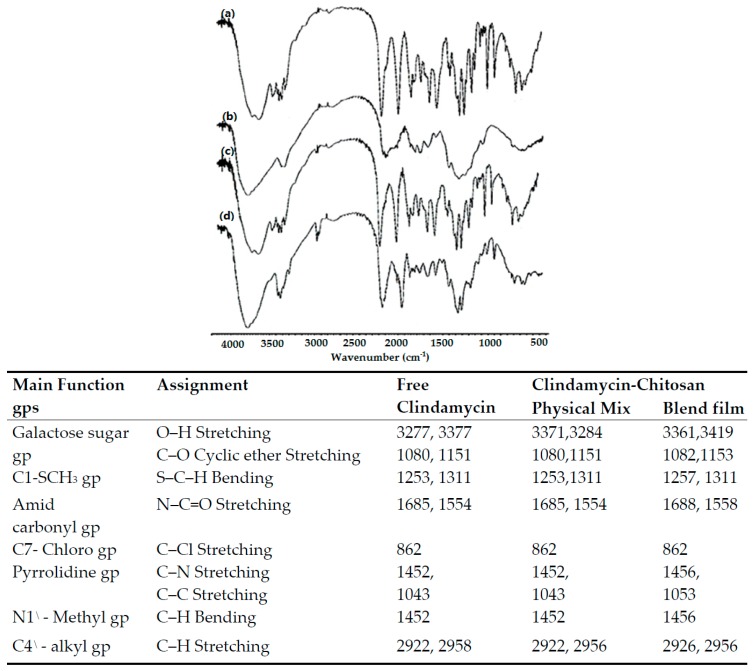
Most effective FTIR bands and spectra of: (a) pure clindamycin; (b) chitosan alone; (c) clindamycin-chitosan physical mixture; (d) clindamycin-chitosan blend film.

In order to investigate drug–polymer chemical interactions, samples were tested by FTIR. The FTIR spectral analysis measures changes in the frequency and bandwidth of interacting groups in the spectrum of the pure drug and polymer that arise when these components are mixed, based on molecular-level changes in oscillation of molecular dipoles [22]. Figure 9 and Figure 10 show the most effective FTIR bands of the drug alone and its polymer blends. The IR spectrum of pure clindamycin shows peaks at the following locations: at 3277 and 3377 cm^−1^ due to O–H stretching in the galactose sugar group [23]; at 1080 and 1151 cm^−1^ due to C–O cyclic ether stretching in the galactose sugar group [24]; at 1253 and 1311 cm^−1^ due to S–C–H bending of C1–SCH_3_ group [25]; at 1685 and 1554 cm^−1^ due to N–C=O stretching of amid carbonyl group [26]; at 862 cm^−1^ due to C–Cl stretching of C7–chloro group [23]; at 1452 cm^−1^ due to C–N stretching of pyrrolidine group; at 1043 cm^−1^ due to C–C stretching of pyrrolidine group; at 1452 cm^−1^ due to C–H bending of N1^\^–methyl group; and finally at 2922 and 2958 cm^−1^ due to C–H stretching of C4^\^–alkyl group [26]. The FTIR spectrum of physical mixture and blend films of clindamycin with both polymers showed no absence of any functional peaks in any spectra, thus revealing that there is no significant chemical interaction between the drug and these polymers. In addition, there were no new bands observed in drug–polymer blends, which confirms that no new chemical bonds were formed between the drug and polymers studied.

### 3.3. Effect of Polymers on Clindamycin Anti-Bacterial Activity against Staphylococcus aureus

The in vitro anti-bacterial activity of clindamycin and its polymer blends was measured by agar dilution susceptibility tests, in which *Staphylococcus aureus* bacterial cells were tested for their ability to produce visible growth on a series of agar plates containing serial dilutions of clindamycin, polymer, or a 1:1 blend. The lowest concentration of each dilution that inhibits the visible growth of *Staphylococcus aureus* was expressed as the MIC. Results obtained were collected in Table 3. For the free drug, the lowest concentration that inhibited visible growth after incubation (MIC) was determined as 4 μg/mL, and this did not change in the clindamycin-alginate or clindamycin-chitosan blends, indicating there is no polymer effect on the anti-microbial activity of the drug.

## 4. Conclusions

Drug–excipient compatibility study has become an indispensable part of the pre-formulation step in the development of new or established drugs. The results suggest that the chemometrics-assisted UV-spectrophotometric analysis could be used as an efficient tool to evaluate compatibility between drugs and excipients. In this research, the recovery studies showed a good compatibility between clindamycin and sodium alginate or chitosan even after seven days’ storage time. Moreover, recovery results suggest a better drug compatibility with an alginate polymer than with a chitosan polymer. The physico-chemical analyses (DSC, XRD, and FTIR) showed no evidence of a strong interaction between clindamycin and either polymer. In addition to these techniques, the in vitro anti-microbial activity of drug against *Staphylococcus aureus* did not change in either the clindamycin-alginate or clindamycin-chitosan blend, indicating their appropriate compatibility.

## Figures and Tables

**Table 1:** Statistical parameters from leave-one out cross validation (CV) of chemometric models for drug–polymer calibration mixtures in the range of 190–250 nm.

Model	PCs	RMSECV	*R*^2^	Offset	Bias
Clindamycin-Alginate Calibration Mixtures	3	0.00284	0.996	−0.0000774	−0.0000338
Clindamycin-Chitosan Calibration Mixtures	2	0.01228	0.942	−0.0052874	−0.0013723

PCs: PLS Principal Components; RMSECV: Root mean square error of cross validation; *R*^2^: Calibration coefficient.

**Table 2:** Recovery studies for clindamycin in (1:1) drug–polymer test mixtures.

Model	Clindamycin Concentration Average Recovery (%) * ± (SD) **		
	Sample	After 3 Days	After 7 Days
**Clindamycin-Alginate Calibration Mixtures**	Pure Clindamycin	99.1 ± 1.4	97.1 ± 0.6
	Clindamycin-Alginate Test Mixtures	98.1 ± 2.9	95.4 ± 4.0
**Clindamycin-Chitosan Calibration Mixtures**	Pure Clindamycin	101.8 ± 1.5	100.6 ± 2.5
	Clindamycin–Chitosan Test Mixtures	90.4 ± 3.0	81.3 ± 4.2
	Clindamycin–Chitosan Test Mixtures after filtration	97.2 ± 2.1	91.3 ± 3.8

* In comparison with concentration at time zero; ** Average of duplicate samples with five concentrations.

**Table 3:** Results of agar dilution susceptibility test.

Sample No.	Composition	MIC (μg/mL)	Remarks
1- Control	Distilled water	-	Effect of solvent
2- Solvent control	0.5% acetic acid	-	Effect of solvent
2- Sodium alginate/water	Sodium alginate/water	-	Effect of polymer
3- Chitosan/water	Chitosan/water	-	Effect of polymer
4- Free Clindamycin	Clindamycin/water	4	Drug activity
5- Clindamycin + Alginate	Clindamycin/Alginate	4	Drug–polymer blending effect
6- Clindamycin + Chitosan	Clindamycin/Chitosan	4	Drug–polymer blending effect
